# Yeast Species, Strains, and Growth Media Mediate Attraction of *Drosophila suzukii* (Diptera: Drosophilidae)

**DOI:** 10.3390/insects10080228

**Published:** 2019-07-31

**Authors:** Rodrigo Lasa, Laura Navarro-de-la-Fuente, Anne C. Gschaedler-Mathis, Manuel R. Kirchmayr, Trevor Williams

**Affiliations:** 1 Red de Manejo Biorracional de Plagas y Vectores, Instituto de Ecología AC, Xalapa, Veracruz 91070, Mexico; 2 Facultad de Ciencias Agrícolas, Universidad Veracruzana, Xalapa, Veracruz 91090, Mexico; 3 Biotecnología Industrial, Centro de Investigación y Asistencia en Tecnología y Diseño del Estado de Jalisco, Zapopan, Jalisco 45019, Mexico

**Keywords:** *Saccharomyces cerevisiae*, *Hanseniaspora uvarum*, *Candida*, *Pichia*, fermentation, sucrose, corn syrup

## Abstract

Specific ecological interactions between insects and microbes have potential in the development of targeted pest monitoring or control techniques for the spotted wing drosophilid, *Drosophila suzukii* (Matsumura), an exotic invasive pest of soft fruit. To evaluate *D. suzukii* attraction to yeast species from preferred types of fruit, three yeasts were isolated from blackberry fruit and two yeasts from raspberry fruit and used to bait simple plastic bottle traps. *Saccharomyces cerevisiae* and *Hanseniaspora uvarum* were identified from blackberries, whereas a different *H. uvarum* strain was identified from raspberry. Yeast identification was based on sequence analysis of the D1/D2 domain of the large subunit 26S rRNA gene. Commercial baker’s yeast (*S. cerevisiae*) was similar or more effective for the capture of *D. suzukii* males and females than yeasts isolated from blackberry or raspberry when grown in sucrose. However, when grown in corn syrup, a strain of *S. cerevisiae* from blackberry captured the highest number of females and a strain of *H. uvarum* isolated from raspberry captured high numbers of males and females. Species of *Candida*, *Hanseniaspora*, and *Pichia* from a laboratory yeast collection did not outperform baker’s yeast in pairwise tests when grown in sucrose solution or yeast-peptone-dextrose medium. The raspberry strain of *H. uvarum* grown in corn syrup outperformed *S. cerevisiae* grown in sucrose, in terms of captures in baited traps under laboratory conditions. We conclude that yeast species, strain, and growth medium can have a marked influence on *D. suzukii* attraction to baited traps, a finding that could assist in the development of yeast-related monitoring or control techniques targeted at this pest.

## 1. Introduction

The spotted wing drosophilid, *Drosophila suzukii* (Matsumura), is an exotic invasive pest that is threatening soft fruit production in many countries around the world, including the Americas and Europe [1,2,3,4]. Specific ecological interactions between insects and microbes could have great potential value in the development of targeted pest monitoring or control techniques [5], and may be applicable to this drosophilid pest [6,7]. A clear association has been established between some yeasts of the family Saccharomycetaceae and drosophilids because (i) yeasts constitute a key nutritional source that improves fitness, sexual maturation, egg production, and courtship performance of adult flies and (ii) dispersal of yeasts by adult flies increases the dissemination and genetic diversity of these microbes [8,9,10,11,12,13]. Indeed, many species of *Drosophila* are attracted to volatile compounds produced by fermenting yeasts, or bacterial metabolites of these compounds [14,15,16,17], a feature that could be exploited for the development of effective and selective attractants for use in crop settings. Of the yeast species associated with *Drosophila,* the majority belong to the genera *Saccharomyces, Hanseniaspora*, *Candida,* and *Pichia*, all within the family Saccharomycetaceae [9,12,18,19].

Trap attractants that have shown promise for trapping *D. suzukii* are often based on fermenting mixtures of *Saccharomyces cerevisiae* and sucrose [20,21,22,23], a yeast that has been selected because it is attractive to *D. suzukii*, cheap, readily available, and grows very well in sucrose solution. Indeed, captures of *D. suzukii* in traps baited with fermenting preparations of *S. cerevisiae* can exceed that of a commonly-used lure based on apple cider vinegar [20,21,22,23]. Another yeast, *Hanseniaspora uvarum*, is closely associated with *D. suzukii* and has been isolated from field-collected flies and larvae, and from the surface of fruit infested by this pest [9]. Attraction of native and exotic drosophilids to fruit inoculated with *H. uvarum* and other yeasts varies across fly species [24], but such responses might be useful in the development of effective and selective attractants for monitoring *D. suzukii* populations, or mass-trapping techniques for pest control [7].

Attraction of adult drosophilids to yeasts is likely to depend on the yeast species [24], the origin of each yeast strain [15], and the growth medium [17,25]. Consequently, in the present study we addressed three issues. First, we isolated and genetically identified yeasts that were present on samples of blackberry and raspberry, which are the preferred hosts of this pest. Second, we determined whether traps baited with fruit-isolated yeasts captured higher numbers of *D. suzukii* in comparison with a commercial strain of *S. cerevisiae* (baker’s yeast) when grown using sucrose or corn syrup. Third, we compared the attraction of *D. suzukii* to other species of *Candida*, *Hanseniaspora*, and *Pichia* from different sources in comparison with *S. cerevisiae* when grown in sucrose or a favorable yeast growth medium (yeast-peptone-dextrose medium). Our studies revealed differences in the attraction of the pest to yeast species and strains, and a marked influence of the growth medium, a feature that could have implications for the use of yeast-based lures targeted at *D. suzukii*.

## 2. Materials and Methods

### 2.1. Insect Colony

A laboratory colony of *D. suzukii* was started at the Instituto de Ecología AC, Xalapa, Mexico, using individuals that emerged from blackberry (*Rubus fruticosus* L.) collected in June 2015 in Xico, Veracruz, Mexico. Adults were allowed to oviposit in a cornmeal-based artificial diet [26], dispensed into 300 mL plastic cups and covered with fine nylon gauze. The colony was maintained at 24 ± 1 °C, 65% ± 10% relative humidity (RH) and 12:12 h (L:D) photoperiod with a light intensity of 3500–4500 lux. Flies used in tests were kept together (both sexes) following emergence in cages and had presumably mated. A moist cotton pad was available in cages as a water supply. A 3:1 mixture of sucrose and inactivated yeast hydrolysate was used to feed adult flies.

### 2.2. Yeast Isolation from Macerated Blackberry and Raspberry Fruits

Blackberry and raspberry (*Rubus idaeus* L.), fruits from Michoacán, Mexico (Driscoll’s, Jacona, Mexico) were acquired from a local supermarket. A group of eight randomly-selected fruits of each species was placed in a previously sterilized ceramic mortar and macerated in 8 mL of sterile distilled water. The resulting homogenate was serially diluted in sterile water and used to inoculate Petri plates containing yeast-peptone-dextrose (YPD; 10 g/L yeast extract, 20 g/L peptone, 20 g/L dextrose) (Dibico, Cuatitlán, Mexico) in 15 g/L agar and 0.5 g/L chloramphenicol to suppress the growth of bacteria. Petri plates were then incubated at 26 °C for 24 h. Single yeast colonies were selected from plates on which low numbers of colonies (20–80 colonies/plate) had grown. Each selected colony was then diluted in 100 µL of sterile water and inoculated on to a new YPD agar + chloramphenicol plate to ensure isolation of pure strains of yeast.

### 2.3. Yeast Identification

Identification of blackberry and raspberry yeast isolates was performed alongside a commercial strain of *S. cerevisiae* (dry baker’s yeast, Tradi-Pan, Safmex SA de CV, Toluca, Mexico), which was also used as a reference treatment in our experiments (designated Tradi-Pan-Sc). Yeasts were grown in 250 mL Erlenmeyer flasks with 50 mL YPD broth inoculated with each isolate and incubated for 48 h at 25 °C in an orbital shaker at 120 rpm. DNA was extracted using an Invitrogen^TM^ PureLink^TM^ Genomic DNA mini-kit. The partial sequences of 26S rDNA were amplified using the universal primer pair NL1 (5′-GCA TAT CAA TAA GCG GAG GAA AAG-3′)/NL4 (5′-GGT CCG TGT TTC AAG ACG G-3′) synthetized by T4 Oligo (Irapuato, Guanajuato, Mexico). PCR products were sequenced using NL1/NL4 primers (Labsergen Sequencing Services, Irapuato, Mexico). The resulting sequences were compared against the corresponding entries of yeast type species in NCBI GenBank [27]. Comparisons of percentage similarity of PCR product nucleotide sequences among isolates and type species were performed using MegAlign (DNASTAR, Madison, WI). All partial 26S rRNA sequences were deposited in NCBI GenBank [27].

### 2.4. Attraction of D. suzukii to Yeast Isolates from Blackberry and Raspberry Fruit

#### 2.4.1. Initial Evaluation of Isolates

**Figure 1:**
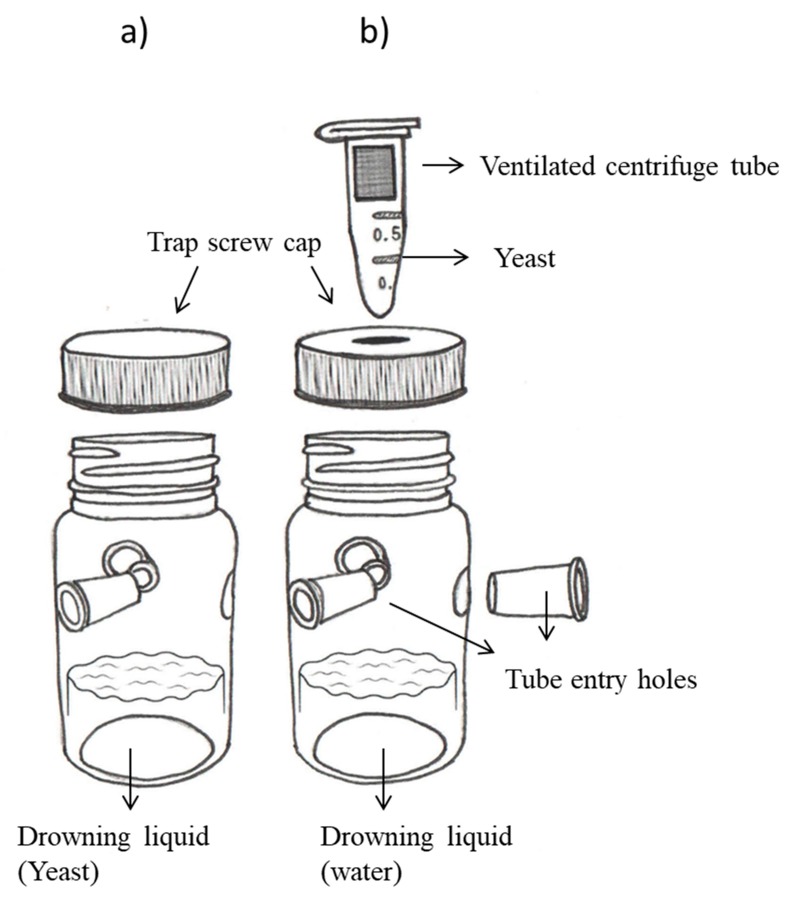
Trap models used for laboratory experiments comprising a 120 mL transparent plastic bottle with (**a**) a conventional white screw cap and (**b**) a white perforated screw cap in which a ventilated centrifuge tube containing yeast samples could be inserted.

Attraction of *D. suzukii* to volatiles of yeasts isolated from blackberry and raspberry were evaluated in pairwise tests, all performed with reference to dry baker’s yeast (Tradi-Pan-Sc). All yeasts, including Tradi-Pan-Sc, were grown in 250 mL Erlenmeyer flasks with 50 mL YPD broth inoculated with ~1 × 10^6^ cells and incubated for 48 h at 25 °C in an orbital agitation shaker at 120 rpm. After growth, yeast cells were counted under an optical microscope (400×) using a hemocytometer (Neubauer Improved, Hawksley, Lancing, United Kingdom). Lures were prepared by inoculating 1.5 × 10^8^ cells of each yeast in 20 mL of (i) 5.5% (wt/vol) sucrose solution or (ii) 5.5% (vol/vol) high fructose corn syrup containing 72.6 g/100 mL of total carbohydrates (Karo bebe^®^, ACH-Foods Mexico, Santa Fé, Mexico). Traps were constructed from 120 mL transparent plastic bottles (35 mm diameter, 87 mm high) that were perforated with three equidistant lateral holes through which translucent conical tubes (9 mm external diameter, 6 mm internal diameter, 20 mm deep) were inserted to decrease the frequency of fly escape once inside the trap. Holes were placed 45 mm above the base of the bottle (Figure 1a).

Pairwise comparisons were performed for Tradi-Pan-Sc and each of the isolated yeasts using 5.5% sucrose solution or 5.5% corn syrup as the growth media. A volume of 20 mL of yeast (1.5 × 10^8^ cells) and growth medium was placed in each trap with 10 µL of Tween 80 as a wetting agent. The mixture was allowed to ferment at 24 °C under laboratory conditions for 24 h prior to use in experiments. Traps were placed at opposite lateral sides of acrylic cages (30 × 30 × 30 cm), and initially assigned to random positions, but the position of each trap was subsequently changed for each replicate. A moist cotton wool wick was present in cages during each experiment as a water source. A group of 40 non-starved, 5-d-old flies (20 females and 20 males) were released inside each cage. All tests were performed under the same conditions used to rear the laboratory insect colony. Flies captured in traps were collected 23 h later, sorted by sex, and counted. The remaining flies inside the cage were discarded. Four independent cages were prepared simultaneously for each test, and traps were evaluated at both positions within each cage, giving eight replicates in total.

#### 2.4.2. Additional Evaluation of RiM2-Hu Isolate from Raspberry

An isolate of *H. uvarum* from raspberry (*R. idaeus*), designated RiM2-Hu, was used for an additional laboratory test based on the results obtained in the previous section. Since the highest captures of flies in the previous tests were observed using the RiM2-Hu isolate grown in corn syrup and Tradi-Pan-Sc grown in sucrose, an additional laboratory test was conducted to compare directly captures in traps baited by each of these yeast + growth medium combinations, using the same methodology described in the previous experiments.

### 2.5. Attraction of D. suzukii to Other Yeast Species

Species from the genera *Hanseniaspora* (*H. lachancei*), *Candida* (*Candida boidinii, Candida glabrata, Candida humilis, Candida tropicalis*), and *Pichia* (*Pichia anomala, Pichia kluyveri, Pichia kudriavzevii, Pichia manshurica, Pichia membranifaciens*) (Table 1), were obtained from the yeast collection held at the Centro de Investigación y Asistencia en Tecnología y Diseño del Estado de Jalisco (CIATEJ), Mexico. Yeasts from this collection were isolated and identified by MALDI-TOF mass spectrometry, as described previously [28]. Attraction of *D. suzukii* to fermenting yeasts was evaluated by capture of flies in pairwise trap tests against the reference Tradi-Pan-Sc strain. As described in the previous section, all yeasts, including Tradi-Pan-Sc, were grown in YPD broth inoculated with ~1 × 10^6^ cells, incubated for 48 h at 25 °C and were counted using a hemocytometer. Two sets of tests were performed using traps that differed from the previous experiments in that a 0.5 mL volume of the yeast + growth media mixture was placed in a 1.5 mL plastic tube with lateral perforations covered with nylon mesh that was inserted into the lid of the trap to prevent contamination of the fermenting mixture from the microorganisms present on flies that entered the trap (Figure 1b).

In the first set of tests, lures were prepared by inoculating 1 × 10^8^ cells of each yeast in 0.5 mL of 5.5% (wt/vol) sucrose solution. Each yeast was inoculated into the medium 2 h before use in tests. In the second set of tests, all yeasts were compared with the reference Tradi-Pan-Sc by taking a 0.5 mL sample directly from the Erlenmeyer flask of YPD media in which the yeast had grown for 48 h at 25 °C, independently of the cell counts of each yeast that varied among yeast species (Supplementary Material, Table S1).

In both sets of experiments, 20 mL water and 10 µL Tween 80 was placed in the bottom of the trap as the drowning solution. Traps were placed on opposite lateral sides of acrylic cages (30 × 30 × 30 cm) initially assigned to random positions that were subsequently switched for each new replicate. All other procedures for cage experiments were identical to those described in Section 2.4. Four independent cages were prepared simultaneously and evaluated at both positions to generate eight replicates.

### 2.6. Statistical Analyses

Mean numbers of trapped males and females of *D. suzukii* in traps baited with yeasts fermented in each growth medium were compared with the reference Tradi-Pan-Sc treatment by paired *t*-test. Mean percentages of total trapped flies in experiments involving sucrose or YPD were compared by *t*-test as percentage values were normally-distributed and did not require transformation. All analyses were performed using the R-based program Jamovi v.0.9.5.17 [29] (Supplementary Material, Tables S2–S5).

## 3. Results

### 3.1. Yeast Identification

Five yeast colonies were selected from YPD-chloramphenicol plates inoculated with fruit homogenate: three originated from blackberry fruit (named using the initials of the plant species and an alphanumerical suffix: RfM1, RfM2, and RfM3) and two from raspberry fruit (coded as RiM1 and RiM2). Sequencing of PCR products (559–565 nt in length) revealed that the partial 26S rDNA sequences of RfM1 corresponded to *S. cerevisiae*, whereas RfM2, RfM3, RiM1, and RiM2, corresponded to *H. uvarum*, with 99.8–100% sequence similarity (Table 2). The baker’s yeast Tradi-Pan-Sc was confirmed as an isolate of *S. cerevisiae* that was identical in the amplified sequence to the *S. cerevisiae* strain isolated from blackberry.

### 3.2. Attraction of D. suzukii to Yeasts Isolated from Blackberry and Raspberry Fruits

**Figure 2:**
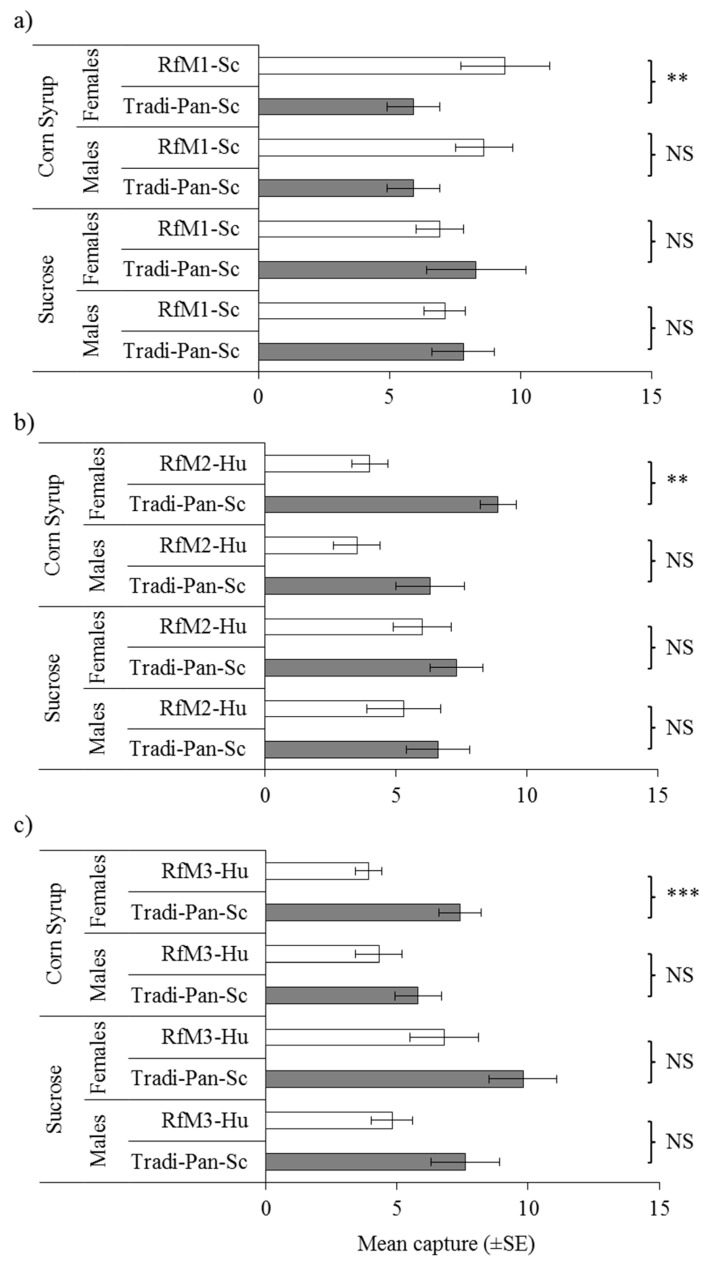
Mean numbers of trapped males and females in pairwise comparisons of blackberry isolated yeasts; (**a**) *Saccharomyces cerevisiae,* RfM1-Sc, (**b**) *Hanseniaspora uvarum*, RfM2-Hu, and (**c**) *H. uvarum*, RfM3-Hu with the reference baker’s yeast *S. cerevisiae* (Tradi-Pan-Sc) when grown in sucrose or corn syrup. NS indicates no significant difference *p* > 0.05; ** denotes significant difference *p* < 0.05; *** *p* < 0.001, (paired *t*-test).

Traps baited with Tradi-Pan-Sc or the *S. cerevisiae* isolate from blackberry, RfM1-Sc, had similar mean captures of both sexes of *D. suzukii* when fermented in sucrose (Figure 2a). Higher numbers of *D. suzukii* females, but not males, were captured in RfM1-Sc baited traps strain when grown in corn syrup, compared to Tradi-Pan-Sc grown in corn syrup (Figure 2a). The opposite pattern was observed in traps baited with Tradi-Pan-Sc or the *H. uvarum* strains from blackberry, RfM2-Hu, and RfM3-Hu (Figure 2b,c). In this case, Tradi-Pan-Sc grown in corn syrup captured significantly higher numbers of *D. suzukii* females than RfM2-Hu or RfM3-Hu, whereas all other comparisons were non-significant (Figure 2b,c).

**Figure 3:**
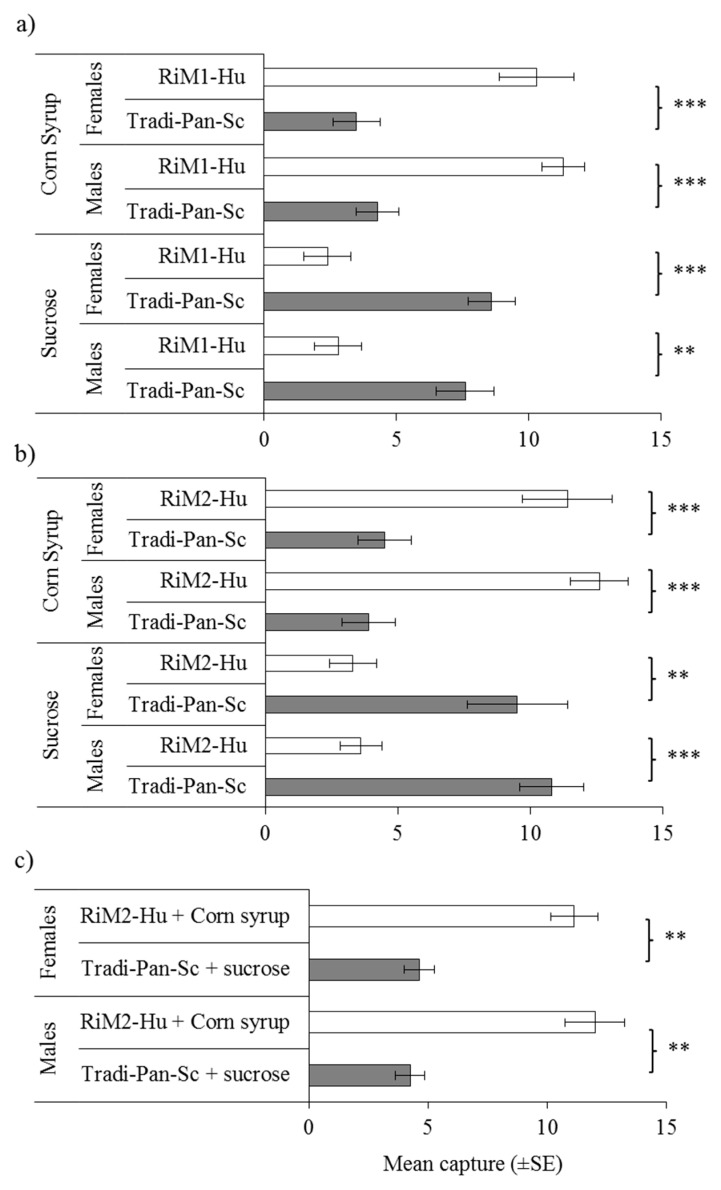
Mean numbers of trapped males and females in pairwise comparisons of raspberry-isolate yeasts (**a**) *Hanseniaspora uvarum*, RiM1-Hu and (**b**) *H. uvarum*, RiM2-Hu with the reference baker’s yeast *Saccharomyces cerevisiae* (Tradi-Pan-Sc) grown in sucrose or corn syrup, or (**c**) *H. uvarum*, RiM2-Hu grown in corn syrup compared with *S. cerevisiae* (Tradi-Pan-Sc) grown in sucrose. ** denotes significant difference *p <* 0.01; *** *p <* 0.001, (paired *t*-test).

The results involving isolates from raspberry were quite different but were consistent among tests (Figure 3a,b). Captures of both sexes of *D. suzukii* in traps baited with the *H. uvarum* isolates from raspberry, RiM1-Hu, and RiM2-Hu, were significantly higher than captures in Tradi-Pan-Sc treated traps when yeasts were fermented in corn syrup. However, when fermented in sucrose solution, captures of both sexes were significantly higher in Tradi-Pan-Sc traps than in RiM1-Hu or RiM2-Hu (Figure 3a,b).

As Tradi-Pan-Sc fermented with sucrose and the raspberry-derived isolates of *H. uvarum* captured the highest numbers of flies in the previous tests (and given that RiM1-Hu and RiM2-Hu were genetically identical in the 26S rDNA region that we analyzed), we performed a direct comparison of traps baited with either RiM2-Hu in corn syrup or Tradi-Pan-Sc in sucrose solution. Traps containing RiM2-Hu + corn syrup had a three-fold higher capture of males and females of *D. suzukii* than traps containing Tradi-Pan-Sc fermented in sucrose solution (Figure 3c).

### 3.3. Attraction of D. suzukii to Species of Candida, Pichia, and Hanseniaspora

Pairwise comparisons were performed between traps baited with Tradi-Pan-Sc and several species of *Candida*, *Pichia*, and *Hanseniaspora* from a yeast research collection, all fermented in sucrose solution (Table 3). Numbers of flies captured in traps baited with *C. boidinii*, *C. glabrata*, *C. humilis, C. tropicalis*, *H. lachancei, P. kudriavzevii*, and *P. membranifaciens* did not differ significantly from Tradi-Pan-Sc baited traps for either sex of *D. suzukii*. In contrast, traps baited with *P. kluyveri* captured significantly fewer males and females than Tradi-Pan-Sc baited traps, whereas traps containing *P. anomala* and *P. manshurica* captured significantly fewer males and females, respectively, compared to Tradi-Pan-Sc baited traps (Table 3).

When yeasts were evaluated in YPD medium, captures of both sexes were similar in traps baited with Tradi-Pan-Sc or *C. boidinii*, *C. glabrata*, *C. humilis, C. tropicalis*, *H. lachancei*, and *P. kudriavzevii* (Table 4). As observed in the previous experiment, traps baited with *P. kluyveri* + YPD had a significantly lower capture of both sexes of *D. suzukii* than Tradi-Pan-Sc + YPD baited traps. In contrast, captures of females were significantly lower in traps baited with *P. anomala*, *P. manshurica*, and *P. membranifaciens* than for Tradi-Pan-Sc baited traps, whereas captures of males did not vary significantly in these tests (Table 4).

Overall captures of *D. suzukii* were similar when yeasts were fermented in YPD or sucrose. Overall, and across all comparisons performed (*n* = 80 comparisons in total), similar mean (±SE) percentages of flies were trapped in experiments involving yeasts in sucrose solution (56.5% ± 1.4%) as yeasts in YPD medium (59.8% ± 1.5%) (*t* = 1.62, df = 158, *p* = 0.108).

## 4. Discussion

Of the five yeasts that we isolated from berry fruits, four were strains of *H. uvarum* and one was a strain of *S. cerevisiae*. Yeast identification was based on sequence analysis of the D1/D2 domain of the large subunit 26S rRNA gene, which is a valuable species indicator in the identification of yeasts [30]. Genetic analysis indicated that the RfM2-Hu and RfM3-Hu isolates from blackberry were genetically indistinguishable isolates of *H. uvarum*, whereas the isolates from raspberry, RiM1-Hu and RiM2-Hu, corresponded to a different strain of *H. uvarum*. Responses of *D. suzukii* to the *H. uvarum* isolates were highly consistent when presented in pairwise comparisons with the commercial strain of *S. cerevisiae* (Tradi-Pan-Sc) in sucrose or corn syrup. The *S. cerevisiae* isolate from blackberry RfM2-Sc was identified as being genetically distinct from commercial baker’s yeast (Tradi-Pan-Sc), which is perhaps unsurprising given their disparate origins.

The attraction of *D. suzukii* to different berry-derived yeast isolates, in comparison to the Tradi-Pan-Sc strain, varied among yeast isolates and according to the fermentation media. In the presence of sucrose, the Tradi-Pan-Sc strain was similar or more effective in the capture of both sexes of *D. suzukii* than almost all the fruit-derived yeasts. As the Tradi-Pan-Sc strain has been selected for the fermentation of bakery products that often contain sucrose [31], so that the growth rate, yeast cell related volatiles, and carbon dioxide production were likely to be higher in this strain than in the natural fruit-derived isolates.

In contrast, the RfM2-Sc isolate from blackberry was significantly more attractive to *D. suzukii* females than Tradi-Pan-Sc in the presence of corn syrup. Similarly, when the *H. uvarum* isolates from raspberry (RiM1-Hu and RiM2-Hu) were grown in corn syrup, higher captures of *D. suzukii* males and females were recorded compared to the Tradi-Pan-Sc strain, contrary to the pattern of captures that we observed for these isolates in the presence of sucrose. Finally, a direct comparison of RiM2-Hu + corn syrup and the commercial Tradi-Pan-Sc strain + sucrose revealed markedly higher captures of both sexes in traps containing the raspberry isolate in combination with corn syrup (Figure 3c).

The ability of different species of yeasts to attract drosophilids often differs according to yeast species and among different strains of the same species [15]. None of the yeast species of *Candida* and *Pichia* or *Hanseniaspora lachancei* evaluated in this study was significantly more attractive than Tradi-Pan-Sc in sucrose or YPD medium. These yeasts were isolated from traditional Mexican drinks based on fermented maize (tejuino) and agave (mescal) and cacao fruit (Table 1), which as habitats for yeasts, differ markedly from the berry fruits used for oviposition by *D. suzukii*. A similar decrease in responses has been observed in *Drosophila melanogaster* when exposed to strains of yeast isolated from non-fruit sources, such as rice, tree bark, and soil [15], whereas flies often respond favorably to strains associated with their host’s habitat [32].

In line with our findings, previous studies identified *H. uvarum* as the most attractive species for *D. suzukii*, particularly when grape juice was used as the fermentation medium [24]. Indeed, *H. uvarum* has been isolated from larvae and adults of *D. suzukii*, as well as fruit infested by this pest [9], although this may not be indicative of an intimate association per se [13]. For example, *Pichia kluyveri* was also reported to be associated with larvae and adults of *D. suzukii* [9], but was significantly less attractive than the commercial Tradi-Pan-Sc strain in our study, a tendency also observed previously [24].

Clearly, growth medium can have a marked effect on the attractiveness of fermenting preparations of yeasts [17]. Appropriate mixtures of nutrients at suitable concentrations are critical for high biomass and the production of volatile compounds in yeasts [33]. Fermentation and the assimilation of carbon sources vary widely across different yeast species [34,35], so that growth medium composition, especially the carbon and nitrogen sources, represent key factors for yeast growth and flavor production [36]. For example, nitrogen availability, and utilization by *S. cerevisiae* significantly influence fermentation kinetics and production of volatile aromatic compounds that are specifically related to the amino acids present in the growth medium [33,37,38,39].

Although yeast species grown in YPD differed significantly in cell counts/mL prior to use in experimental traps (Supplementary Material Table S1), growth curves, and the production of volatiles were not quantified, which should be the subject of future study given the differences in responses that we observed. The *H. uvarum* (RiM2-Hu) + corn syrup combination captured significantly more *D. suzukii* flies than Tradi-Pan-Sc + sucrose under laboratory conditions. In previous experiments, captures of *D. suzukii* in traps baited with undefined strains of *H. uvarum* or *S. cerevisiae* in sucrose solution did not differ significantly in tests performed in blueberry and cherry crops [40]. In this respect, we believe that the complex environment of greenhouse berry crop production, with a complex interaction of high levels of volatiles and variable yeast growth conditions, could influence the attraction to particular yeast strains in comparison with the reference *S. cerevisiae* treatment. As the association of *D. suzukii* with *H. uvarum* is not species-specific, and other drosophilid flies can also be attracted to this yeast [15,24,41], future tests under berry crop conditions in commercial greenhouses are required to determine the efficacy and selectivity of these strains against *D. suzukii*.

## 5. Conclusions

We conclude that in addition to yeast species and strain, growth media is a key factor that determines the attraction of *D. suzukii* flies to a specific yeast preparation. Attraction to fermenting yeasts in traps could potentially be improved by the selection of specific growth media or the addition of specific components to the growth medium. In general, traps baited with a commercial preparation of baker’s yeast (*S. cerevisiae*) in sucrose resulted in consistently high captures of *D. suzukii*, but a raspberry-derived strain of *H. uvarum* and a blackberry-derived strain of *S. cerevisiae* could be potentially more attractive, or provide more selective captures of this pest when fermented with corn syrup. These laboratory-based findings require confirmation in berry crops under commercial production conditions.

## Figures and Tables

**Table 1:** Yeast species used in experiments including the identification code used in this study, source, origin, and GenBank accession number of partial 26S rDNA sequences.

Code	Species	Source ^a^	Origen	Accession No.
Tradi-Pan	*Saccharomyces cerevisiae*	Dry baker’s yeast	Safmex SA de CV	MN153007
RfM1	*Saccharomyces cerevisiae*	Blackberry	Isolated	MN153005
RfM2	*Hanseniaspora uvarum*	Blackberry	Isolated	MN153006
RfM3	*Hanseniaspora uvarum*	Blackberry	Isolated	MN153004
RiM1	*Hanseniaspora uvarum*	Raspberry	Isolated	MN153010
RiM2	*Hanseniaspora uvarum*	Raspberry	Isolated	MN153008
5HL	*Hanseniaspora lachancei*	Mescal	Collection CIATEJ	-
1CB	*Candida boidinii*	Tejuino	Collection CIATEJ	-
2CG	*Candida glabrata*	Tejuino	Collection CIATEJ	-
3CH	*Candida humilis*	Tejuino	Collection CIATEJ	-
4 CT	*Candida tropicalis*	Tejuino	Collection CIATEJ	-
6PA	*Pichia anomala*	Mescal	Collection CIATEJ	-
7PKL	*Pichia kluyveri*	Mezcal	Collection CIATEJ	-
8PKU	*Pichia kudriavzevii*	Cacao	Collection CIATEJ	-
9PMA	*Pichia manshurica*	Mescal	Collection CIATEJ	-
10PME	*Pichia membranifaciens*	Mescal	Collection CIATEJ	-

^a^ Tejuino is a traditional Mexican drink made from fermented maize. Mescal is a distilled drink made from fermented agave plants. All fruit-derived yeasts originated from Mexico.

**Table 2:** Comparison of percentage similarity of PCR-amplified 26S nucleotide sequences of the yeasts isolated in this study and the corresponding type species.

Species and Code	1	2	3	4	5	6	7	8
1. *Saccharomyces cerevisiae* KC881066.1 *	-	85.7	100	100	85.9	85.9	85.7	85.7
2. *Hanseniaspora uvarum* KY107844.1 *		-	85.7	85.7	99.8	99.8	100	100
3. *S. cerevisiae* Tradi-pan			-	100	85.9	85.9	85.7	85.7
4. *S. cerevisiae* RfM1				-	85.9	85.9	85.7	85.7
5. *H. uvarum* RfM2					-	100	99.8	99.8
6. *H. uvarum* RfM3						—	99.8	99.8
7. *H. uvarum* RiM1							—	100
8. *H. uvarum* RiM2								—

* Sequences of type species in GenBank. Numbers above columns (1–8) correspond to numbers in rows (1–8) for comparisons.

**Table 3:** Mean numbers of trapped males and females in pairwise comparisons of the reference yeast *Saccharomyces cerevisiae* (Tradi-Pan-Sc) with different species of *Candida*, *Hanseniaspora*, and *Pichia* grown in sucrose solution.

	Mean ± SE			
Yeasts	Males		Females	
*Saccharomyces cerevisiae*	8.3 ± 1.6	NS	7.3 ± 1.5	NS
*Candida boidinii*	6.9 ± 1.6		6.8 ± 1.6	
*S. cerevisiae*	7.3 ± 1.0	NS	7.9 ± 1.3	NS
*Candida glabrata*	4.1 ± 1.6		4.1 ± 1.3	
*S. cerevisiae*	4.0 ± 1.1	NS	4.6 ± 0.9	NS
*Candida humilis*	5.3 ± 0.9		5.8 ± 1.2	
*S. cerevisiae*	4.9 ± 0.9	NS	6.0 ± 1.0	NS
*Candida tropicalis*	8.1 ± 1.2		5.8 ± 1.1	
*S. cerevisiae*	3.6 ± 0.9	NS	4.5 ± 0.9	NS
*Hanseniaspora lachancei*	3.0 ± 0.5		3.0 ± 0.8	
*S. cerevisiae*	7.6 ± 1.0	*	8.9 ± 1.5	NS
*Pichia anomala*	3.5 ± 0.6		5.3 ± 1.0	
*S. cerevisiae*	9.6 ± 0.8	***	8.6 ± 0.7	***
*Pichia kluyveri*	1.1 ± 0.6		2.1 ± 0.7	
*S. cerevisiae*	3.6 ± 0.9	NS	6.6 ± 0.4	NS
*Pichia kudriavzevii*	4.4 ± 0.9		4.3 ± 1.2	
*S. cerevisiae*	6.8 ± 1.4	NS	7.9 ± 1.2	*
*Pichia manshurica*	3.1 ± 0.5		3.0 ± 0.6	
*S. cerevisiae*	6.1 ± 0.8	NS	7.4 ± 1.3	NS
*Pichia membranifaciens*	5.0 ± 0.4		3.4 ± 1.0	

NS denotes no significant difference *p >* 0.05; * *p <* 0.05; *** *p <* 0.001 (paired *t*-test).

**Table 4:** Mean numbers of trapped males and females in laboratory pairwise comparisons of the reference yeast *Saccharomyces cerevisiae* (Tradi-Pan-Sc) with different species of *Candida*, *Hanseniaspora*, and *Pichia* grown in yeast-peptone-dextrose (YPD) medium.

	Mean ± SE			
Yeasts	Males		Females	
*Saccharomyces cerevisiae*	8.3 ± 1.6	NS	7.3 ± 1.5	NS
*Candida boidinii*	6.9 ± 1.6		6.8 ± 1.6	
*S. cerevisiae*	5.9 ± 1.0	NS	5.3 ± 0.8	NS
*Candida glabrata*	6.3 ± 0.6		4.9 ± 1.1	
*S. cerevisiae*	5.3 ± 1.4	NS	5.1 ± 0.7	NS
*Candida humilis*	4.9 ± 1.2		5.0 ± 1.2	
*S. cerevisiae*	5.1 ± 0.9	NS	4.6 ± 1.4	NS
*Candida tropicalis*	7.5 ± 1.7		6.1 ± 1.5	
*S. cerevisiae*	7.3 ± 1.4	NS	6.8 ± 0.5	NS
*Hanseniaspora lachancei*	4.6 ± 0.9		5.4 ± 0.8	
*S. cerevisiae*	6.6 ± 1.2	NS	9.9 ± 0.6	*
*Pichia anomala*	4.6 ± 0.8		5.5 ± 0.8	
*S. cerevisiae*	9.1 ± 0.7	*	9.3 ± 0.9	*
*Pichia kluyveri*	3.6 ± 0.8		2.9 ± 0.6	
*S. cerevisiae*	4.8 ± 1.0	NS	6.5 ± 0.8	NS
*Pichia kudriavzevii*	3.6 ± 0.7		4.5 ± 0.9	
*S. cerevisiae*	7.5 ± 1.2	NS	10.5 ± 0.9	***
*Pichia manshurica*	3.4 ± 1.0		3.0 ± 0.6	
*S. cerevisiae*	7.0 ± 1.3	NS	8.5 ± 1.3	*
*Pichia membranifaciens*	2.5 ± 0.8		2.6 ± 0.9	

NS denotes a non-significant difference *p* > 0.05; * *p* < 0.05; *** *p* < 0.001, (paired *t*-test).

## Supplementary Materials

The following are available online at https://www.mdpi.com/2075-4450/10/8/228/s1, Table S1: Growth of yeast species in YPD medium, Table S2: Statistical analyses applied to results in Figure 2, Table S3: Statistical analyses applied to results in Figure 3, Table S4: Statistical analyses applied to results in Table 3, Table S5: Statistical analyses applied to results in Table 4.
