# Supplementary material for: Yeast Species, Strains, and Growth Media Mediate Attraction of Drosophila suzukii (Diptera: Drosophilidae)
Source: Insects. 2019 Jul 31;10(8):228. doi: 10.3390/insects10080228 (PMC6722520; doi:10.3390/insects10080228)
Supplement: Supplementary file 1 [file insects-10-00228-s001.zip › Lasa_suppl_flies/Table S1.pdf]

Table S1. Growth of yeast species in YPD medium.

**Yeast species**	**Cells/ml (± SE)**	
C. glabrata	$1.7 \pm 0.2 \times 10^9$	**a**
P. menbranifaciens	$1.3 \pm 0.8 \times 10^9$	**ab**
C. humilis	$1.2 \pm 0.2 \times 10^9$	**b**
C. tropicalis	$1.2 \pm 0.4 \times 10^9$	**abc**
P. kudriavzevii	$1.1 \pm 0.1 \times 10^9$	**bc**
P. manshurica	$1.1 \pm 0.1 \times 10^9$	**bc**
P. anomala	$9.9 \pm 2.0 \times 10^8$	**bc**
P. kluyveri	$9.1 \pm 0.9 \times 10^8$	**bc**
H. lachancei	$9.0 \pm 1.5 \times 10^8$	**bc**
C. boidinii	$5.6 \pm 0.3 \times 10^8$	**c**
S. cerevisiae (Tradi-Pan)	$4.3 \pm 0.2 \times 10^8$	**d**

Mean growth of different yeast species following inoculation of ~1 $x10^6$ cells in 50 ml of yeast-peptone dextrose (YPD) broth and incubation at 25 °C during 48 h at 120 rpm orbital agitation. Three replicates were performed. Values followed by different letters indicate statistical differences (ANOVA, Tukey, $p<0.05$).
