# Supplementary material for: Yeast Species, Strains, and Growth Media Mediate Attraction of Drosophila suzukii (Diptera: Drosophilidae)
Source: Insects. 2019 Jul 31;10(8):228. doi: 10.3390/insects10080228 (PMC6722520; doi:10.3390/insects10080228)
Supplement: Supplementary file 1 [file insects-10-00228-s001.zip › Lasa_suppl_flies/Table S2.pdf]

Table S2. Statistical analyses applied to results in Figure 2.

The t-test values applied to results in Figure 2a,b,c in terms of means, difference between means, SE of that difference for each sex of *D. suzukii*.

a)	Yeast	Males ± SE		Student t-test	Females ± SE		Student t-test
Sucrose	Tredi-Pan-Sc	7.1 ± 0.7	NS	t=0.820; df=15; p=0.425; Mean diff=0.750; SE diff=0.915	7.0 ± 1.0	NS	t=0.716; df=15; p=485; Mean diff=0.1.06; SE diff=1.48
	RfM1-Sc	6.4 ± 0.6			8.1 ± 0.7		
Corn syrup	Tredi-Pan-Sc	5.1 ± 0.6	NS	t=1.51; df=15; p=0.151; Mean diff=1.75; SE diff=1.16	5.6 ± 0.6	**	t=2.23; df=15; p=0.041; Mean diff=2.88; SE diff=1.29
	RfM1-Sc	6.8 ± 0.8			8.5 ± 0.9		

b)	Yeast	Males ± SE		Student t-test	Females ± SE		Student t-test
Sucrose	Tredi-Pan-Sc	6.6 ± 1.2	NS	t=0.544; df=7; p=0.603; Mean diff=1.38; SE diff=2.53	7.3 ± 1.0	NS	t=0.629; df=7; p=0.550; Mean diff=1.25; SE diff=1.99
	RfM2-Hu	5.3 ± 1.4			6.0 ± 1.1		
Corn syrup	Tredi-Pan-Sc	6.3 ± 1.3	NS	t=1.40; df=7; p=0.204; Mean diff=2.75; SE diff=1.96	8.9 ± 0.7	**	t=3.75; df=7; p=0.007; Mean diff=4.88; SE diff=1.30
	RfM2-Hu	3.5 ± 0.9			4.0 ± 0.7		

c)	Yeast	Males ± SE		Student t-test	Females ± SE		Student t-test
Sucrose	Tredi-Pan-Sc	7.6 ± 1.4	NS	t=1.81; df=7; p=0.113; Mean diff=2.08; SE diff=1.59	9.8 ± 1.3	NS	t=1.48; df=7; p=0.182; Mean diff=3.00; SE diff=2.03
	RfM3-Hu	4.8 ± 0.8			6.8 ± 1.3		
Corn syrup	Tredi-Pan-Sc	5.8 ± 0.9	NS	t=0.887; df=7; p=0.404; Mean diff=1.50; SE diff=1.69	7.4 ± 0.8	**	t=5.86; df=7; p<0.001; Mean diff=3.50; SE diff=0.598
	RfM2-Hu	4.3 ± 0.9			3.9 ± 0.5		

Mean diff. indicates difference between means; SE diff. indicates standard error of that difference.
