# Supplementary material for: Yeast Species, Strains, and Growth Media Mediate Attraction of Drosophila suzukii (Diptera: Drosophilidae)
Source: Insects. 2019 Jul 31;10(8):228. doi: 10.3390/insects10080228 (PMC6722520; doi:10.3390/insects10080228)
Supplement: Supplementary file 1 [file insects-10-00228-s001.zip › Lasa_suppl_flies/Table S3.pdf]

Table S3. Statistical analyses applied to results in Figure 3.

The t-test values applied to results in Figure 3a,b,c in terms of means, difference between means, SE of that difference for each sex of *D. suzukii*.

**a)**	**Yeast**	**Males ± SE**		**Student t-test**	**Females ± SE**		**Student t-test**
Sucrose	Tredi-Pan-Sc	7.6 ± 1.1	**	t=2.88; df=7; p=0.024; Mean diff=4.88; SE diff=1.69	8.6 ± 0.9	***	t=5.87; df=7; p<0.001; Mean diff=6.25; SE diff=1.06
	RiM1-Hu	2.8 ± 0.9			2.4 ± 0.9		
Corn syrup	Tredi-Pan-Sc	4.3 ± 0.8	***	t=5.24; df=7; p=0.001; Mean diff=7.00; SE diff=1.34	3.5 ± 0.9	**	t=3.32; df=7; p=0.013; Mean diff=6.45; SE diff=2.03
	RiM1-Hu	11..3 ± 0.8			10.3 ± 1.4		
**b)**	**Yeast**	**Males ± SE**		**Student t-test**	**Females ± SE**		**Student t-test**
Sucrose	Tredi-Pan-Sc	10.8 ± 1.0	**	t=4.49; df=7; p=0.003; Mean diff=7.13; SE diff=1.59	9.5 ± 1.4	**	t=3.35; df=7; p=0.012; Mean diff=6.25; SE diff=1.87
	RiM2-Hu	3.6 ± 0.7			3.3 ± 0.6		
Corn syrup	Tredi-Pan-Sc	3.9 ± 0.5	***	t=5.15; df=7; p=0.001; Mean diff=8.75; SE diff=1.70	4.5 ± 0.5	***	t=5.07; df=7; p=0.001; Mean diff=6.88; SE diff=1.36
	RiM2-Hu	12.6 ± 1.3			11.4 ± 1.1		
**c)**	**Yeast**	**Males ± SE**		**Student t-test**	**Females ± SE**		**Student t-test**
Sucrose	Tredi-Pan-Sc	4.3 ± 0.6	**	t=4.98; df=7; p=0.002; Mean diff=7.75; SE diff=1.56	4.6 ± 0.6	**	t=4.23; df=7; p=0.004; Mean diff=6.50; SE diff=1.54
Corn syrup	RiM2-Hu	12.0 ± 1.4			11.1 ± 1.0		
Mean diff. indicates difference between means; SE diff. indicates standard error of that difference.							
