# Supplementary material for: Yeast Species, Strains, and Growth Media Mediate Attraction of Drosophila suzukii (Diptera: Drosophilidae)
Source: Insects. 2019 Jul 31;10(8):228. doi: 10.3390/insects10080228 (PMC6722520; doi:10.3390/insects10080228)
Supplement: Supplementary file 1 [file insects-10-00228-s001.zip › Lasa_suppl_flies/Table S4.pdf]

Table S4. Statistical analyses applied to results in Table 3.

The t-test values applied to results in Table 3 in terms of means, difference between means, SE of that difference for each sex of *D. suzukii*. In all cases yeasts were grown in 5.5% sucrose solution.

Yeast	Mean males ± SE		Student t-test	Mean females ± SE		Student t-test
S. cerevisiae	6.5 ± 0.7	NS	t=0.646; df=7; p=0.539; Mean diff=0.875; SE diff=1.36	6.6 ± 0.9	NS	t=1.51; df=7; p=0.174; Mean diff=2.0; SE diff=1.32
C. boidinii	5.6 ± 1.1			4.6 ± 0.6		
S. cerevisiae	7.3 ± 1.0	NS	t=1.28; df=7; p=0.242; Mean diff=3.13; SE diff =2.45	7.9 ± 1.3	NS	t=1.65; df=7; p=0.143; Mean diff=3.75; SE diff=2.27
C. glabrata	4.1 ± 1.6			4.1 ± 1.3		
S. cerevisiae	4.0 ± 1.1	NS	t=1.23; df=7; p=0.258; Mean diff=1.38; SE diff=1.12	4.6 ± 0.9	NS	t=0.609; df=7; p=0.562; Mean diff=1.13; SE diff=1.85
C. humilis	5.4 ± 0.9			5.8 ± 1.2		
S. cerevisiae	4.9 ± 0.9	NS	t=1.76; df=7; p=0.122; Mean diff=3.25; SE diff=1.85	6.0 ± 1.0	NS	t=0.157; df=7; p=0.879; Mean diff=0.250; SE diff=1.59
C. tropicalis	8.1 ± 1.2			5.8 ± 1.1		
S. cerevisiae	3.6 ± 0.9	NS	t=0.478; df=7; p=0.647; Mean diff=0.625; SE diff=1.31	4.5 ± 0.9	NS	t=0.728; df=7; p=0.490; Mean diff=0.875; SE diff=1.20
H. lachancei	3.0 ± 0.5			3.6 ± 0.8		
S. cerevisiae	7.6 ± 1.0	**	t=2.56; df=7; p=0.037; Mean diff=4.13; SE diff=1.61	8.9 ± 1.5	NS	t=1.49; df=7; p=0.180; Mean diff=3.63; SE diff=2.43
P. anomala	3.5 ± 0.6			5.3 ± 1.0		
S. cerevisiae	9.6 ± 0.8	***	t=6.12; df=7; p<0.001; Mean diff=8.50; SE diff=1.39	8.6 ± 0.7	***	t=5.73; df=7; p<0.001; Mean diff=6.50; SE diff=1.13
P. kluyveri	1.1 ± 0.6			2.1 ± 0.7		
S. cerevisiae	3.6 ± 0.9	NS	t=0.557; df=7; p=0.597; Mean diff=0.750; SE diff=1.35	6.6 ± 0.4	NS	t=1.74; df=7; p=0.125; Mean diff=2.38; SE diff=1.36
P. kudriavzevii	4.4 ± 0.9			4.3 ± 1.2		
S. cerevisiae	6.8 ± 1.4	NS	t=2.12; df=7; p=0.072; Mean diff=3.63; SE diff=1.71	7.9 ± 1.2	**	t=3.74; df=7; p=0.007; Mean diff=5.38; SE diff=1.44
P. manshurica	3.1 ± 0.5			3.0 ± 0.6		
S. cerevisiae	6.1 ± 0.8	NS	t=1.06; df=7; p=0.324; Mean diff=1.13; SE diff=1.06	7.4 ± 1.3	NS	t=1.92; df=7; p=0.097; Mean diff=4.00; SE diff=2.09
P. membranifaciens	5.0 ± 0.4			3.4 ± 1.0		

Mean diff. indicates difference between means; SE diff. indicates standard error of that difference.
