# Supplementary material for: Yeast Species, Strains, and Growth Media Mediate Attraction of Drosophila suzukii (Diptera: Drosophilidae)
Source: Insects. 2019 Jul 31;10(8):228. doi: 10.3390/insects10080228 (PMC6722520; doi:10.3390/insects10080228)
Supplement: Supplementary file 1 [file insects-10-00228-s001.zip › Lasa_suppl_flies/Table S5.pdf]

Table S5. Statistical analyses applied to results in Table 4.

The t-test values applied to results in Table 4 in terms of means, difference between means, SE of that difference for each sex of *D. suzukii*. In all cases yeasts were grown in YPD medium.

Yeast	Mean males ± SE		Student t-test	Mean females ± SE		Student t-test
S. cerevisiae	8.3 ± 1.6	NS	t=0.443; df=7; p=0.671; Mean diff=1.38; SE diff=3.10	7.3 ± 1.5	NS	t=0.171; df=7; p=0.865; Mean diff=0.5; SE diff=2.83
C. boidinii	6.9 ± 1.6			6.8 ± 1.5		
S. cerevisiae	5.9 ± 1.0	NS	t=0.275; df=7; p=0.791; Mean diff=0.37; SE diff=1.36	5.3 ± 0.8	NS	t=222; df=7; p=0.831;Mean diff=0.37; SE diff=1.69
C. glabrata	6.3 ± 0.6			4.9 ± 1.1		
S. cerevisiae	5.3 ± 1.4	NS	t=0.158; df=7; p=0.879; Mean diff=0.37; SE diff=2.37	5.1 ± 0.7	NS	t=0.076; df=7; p=0.941; Mean diff=0.125; SE diff=1.64
C. humilis	4.9 ± 1.2			5.0 ± 1.2		
S. cerevisiae	5.1 ± 0.9	NS	t=1.11; df=7; p=0.305; Mean diff=2.38; SE diff=2.15	4.6 ± 1.4	NS	t=0.580; df=7; p=0.580; Mean diff=1.50; SE diff=2.58
C. tropicalis	7.5 ± 1.7			6.1 ± 1.5		
S. cerevisiae	7.3 ± 1.4	NS	t=1.26; df=7; p=0.249; Mean diff=2.63; SE diff=2.09	6.8 ± 0.5	NS	t=1.46; df=7; p=0.188; Mean diff=1.38; SE diff=0.944
H. lachancei	4.6 ± 0.9			5.4 ± 0.8		
S. cerevisiae	6.6 ± 1.2	NS	t=1.30; df=7; p=0.234; Mean diff=2.00; SE diff=1.54	9.9 ± 0.6	**	t=4.46; df=7; p=0.003; Mean diff=4.38; SE diff=0.981
P. anomala	4.6 ± 0.8			5.5 ± 0.8		
S. cerevisiae	9.1 ± 0.7	**	t=4.66; df=7; p=0.002; Mean diff=5.50; SE diff=1.18	9.3 ± 0.9	**	t=4.68; df=7; p=0.002; Mean diff=6.38; SE diff=1.36
P. kluyveri	3.6 ± 0.8			2.9 ± 0.6		
S. cerevisiae	4.9 ± 1.0	NS	t=0.835; df=7; p=0.431; Mean diff=1.25; SE diff=1.50	6.5 ± 0.8	NS	t=1.30; df=7; p=0.234; Mean diff=2.00; SE diff=1.54
P. kudriavzevii	3.6 ± 0.7			4.5 ± 0.9		
S. cerevisiae	7.5 ± 1.2	NS	t=2.25; df=7; p=0.059; Mean diff=4.25; SE diff=1.89	10.5 ± 0.9	***	t=6.61; df=7; p<0.001; Mean diff=8.00; SE diff=1.21
P. manshurica	3.3 ± 1.0			3.0 ± 0.6		
S. cerevisiae	7.0 ± 1.4	NS	t=2.35; df=7; p=0.051; Mean diff=4.50; SE diff=1.92	8.5 ± 1.4	**	t=3.58; df=7; p=0.009; Mean diff=5.88; SE diff=1.64
P. membranifaciens	2.5 ± 0.8			2.6 ± 0.9		

Mean diff. indicates difference between means; SE diff. indicates standard error of that difference.
